# Emerging priorities for HIV service delivery

**DOI:** 10.1371/journal.pmed.1003028

**Published:** 2020-02-14

**Authors:** Nathan Ford, Elvin Geng, Tom Ellman, Catherine Orrell, Peter Ehrenkranz, Izukanji Sikazwe, Andreas Jahn, Miriam Rabkin, Stephen Ayisi Addo, Anna Grimsrud, Sydney Rosen, Isaac Zulu, William Reidy, Thabo Lejone, Tsitsi Apollo, Charles Holmes, Ana Francisca Kolling, Rosina Phate Lesihla, Huu Hai Nguyen, Baker Bakashaba, Lastone Chitembo, Ghion Tiriste, Meg Doherty, Helen Bygrave

**Affiliations:** 1 Department HIV & Global Hepatitis Programme, World Health Organization, Geneva, Switzerland; 2 Centre for Infectious Disease Epidemiology and Research, School of Public Health and Family Medicine, Faculty of Health Sciences, University of Cape Town, South Africa; 3 Center for Dissemination and Implementation, Institute for Public Health, Washington University, St. Louis, Missouri, United States of America; 4 Southern African Medical Unit, Médecins Sans Frontières, Cape Town, South Africa; 5 Department of Medicine, Faculty of Health Sciences, Cape Town, South Africa; 6 Bill and Melinda Gates Foundation, Seattle, Washington, United States of America; 7 Centre for Infectious Disease Research in Zambia, Lusaka, Zambia; 8 Ministry of Health, Lilongwe, Malawi; 9 ICAP, Columbia University Mailman School of Public Health, New York, New York, United States of America; 10 National AIDS Control Programme, Ministry of Health, Accra, Ghana; 11 International AIDS Society, Cape Town, South Africa; 12 Department of Global Health, Boston University School of Public Health, Boston, Massachusetts, United States of America; 13 Division of Global HIV & TB, Centers for Disease Control and Prevention, Atlanta, Georgia, United States of America; 14 SolidarMed, Swiss Organization for Health in Africa, Butha-Buthe, Lesotho; 15 Ministry of Health and Child Care Zimbabwe, Harare, Zimbabwe; 16 Georgetown University, Washington, DC, United States of America; 17 Department of Surveillance, Prevention and Control of STIs, HIV/AIDS and Viral Hepatitis, Ministry of Health, Brasilia, Brazil; 18 National AIDS Control Programme, Ministry of Health, Maseru, Lesotho; 19 Treatment and Care Department, Viet Nam Authority of HIV/AIDS Control, Ministry of Health, Hanoi, Vietnam; 20 The AIDS Support Organization (TASO), Kampala, Uganda; 21 Department HIV, World Health Organization Lusaka, Zambia; 22 Department HIV, World Health Organization, Addis Ababa, Ethiopia

## Abstract

Nathan Ford and co-authors discuss global priorities in the provision of HIV prevention and treatment services.

**Summary points**

- According to the Joint United Nations Programme on HIV/AIDS (UNAIDS), in 2018, an estimated 37.9 million people were living with HIV worldwide. There were also 1.7 million new infections and 770,000 deaths.
- At the end of June 2019, 24.5 million people were receiving antiretroviral therapy (ART). Nonetheless, increased access to high-quality ART services is needed to further reduce mortality and new infections and to optimize long term outcomes.
- In this article, we summarize priorities for HIV service delivery research and guidance identified through a World Health Organization (WHO) consultation held at the end of 2018.
- The priorities identified include linkage from HIV testing to care; rapid initiation of ART (including out-of-facility ART initiation); task sharing and decentralization, including children and patients on second line; ART delivery for stable clients; adherence, retention, and reengagement in care; management of advanced HIV disease; provision of welcoming health services; and strengthening of service integration, particulary for NCDs and family planning.
- Ongoing evaluation is needed to determine the net effects of introducing differentiated service delivery models, in terms of health service inputs and long-term outcomes for people living with HIV.

## Introduction

To support delivery of antiretroviral therapy (ART) at scale, the World Health Organization (WHO) has promoted a public health approach to HIV treatment and care. Recognizing the critical importance of streamlined, standardized approaches to scaling up HIV services in settings with limited resources, the public health approach emphasizes strategies such as task sharing, decentralization and integration of HIV services with other public health programs, and patient and community empowerment. The public health approach also prioritizes streamlined clinical and laboratory monitoring, standardized first- and second-line treatment regimens, and harmonized monitoring and evaluation strategies [1,2]. In high-income countries with more resources and fewer HIV cases a more individualized approach to HIV care is favored, although the overarching framework of the public health approach provides the setting within which this more personalized service delivery can occur.

Since 2013, WHO has developed evidence-based recommendations aimed at addressing the key steps in the HIV care cascade from HIV testing and linkage to care to initiation of ART and adherence and retention [3]. WHO’s public health approach includes differentiated service delivery, which tailors delivery strategies to specific populations to optimize quality, satisfaction, and efficiency [4]. By responding to patient preferences, needs, and values, differentiated service delivery improves the quality of health systems [5,6].

## Methods

In November 2018, WHO held an expert consultation on future priorities for HIV treatment service delivery. The consultation included national HIV program managers from 11 countries, implementation partners, and representatives from civil society and academia. To support this consultation, WHO conducted a survey to assess the implementation status of current guideline recommendations (S1 Text). Information on the implementation status of service delivery recommendations was accessed from the Joint United Nations Programme on HIV/AIDS (UNAIDS) database http://lawsandpolicies.unaids.org/.

We discuss the findings of the consultation for implementing key service delivery interventions and priorities for future guidance (Table 1). Priorities for future guidance have been grouped according to the HIV care cascade: linkage from HIV testing to care; initiating and maintaining ART; adherence, retention, and reengagement in care; managing advanced HIV disease; and service integration.

**Table 1:** Evolution of HIV service delivery guidance.

Issue	Current guidance	Additional guidance needed
**Linkage from HIV testing to care and prevention**	Peer support and navigation, service integration, and tracing.	Phased strategies of testing distribution and linkage through peers.
		Linkage to prevention.
**Rapid ART initiation**	Within 7 days with the offer of same-day ART.	Cointerventions that maximize treatment outcomes following rapid ART initiation, contextual factors that could influence success of such cointerventions.
		Minimum counseling intervention required prior to and after same-day ART.
		Guidance on timing of ART initiation for settings with differing resources and access to baseline diagnostics related to advanced disease screening.
**Out-of-facility ART initiation**	None.	Out-of-facility ART initiation and cointerventions, duration of community follow-up.
**Adherence and retention in care**	Peer counseling, mobile text messaging, reminder devices, CBT, behavior skills training, fixed-dose combinations.	Optimal approach to measuring adherence, especially as patients in less intense models of care have less interaction with the healthcare system. Timing and frequency of interventions, healthcare worker attitude, patient experience.
	Peer support, adherence clubs, and extra care for high-risk people.	Role of peers (adolescents and key populations).
**Tracing and return to care**	None.	Interventions to support tracing (including frequency and methods) and timely reengagement to care, including nonjudgmental approaches.
**Task sharing and decentralization**	Nurse initiation of ART.	Nurse initiation of ART including for children and regimen change.
	Lay-worker provision of certain essential diagnostic tests.	Defined list of diagnostic tests that can be provided by lay workers with emphasis on the importance of using the result to change patient management.
**Frequency of clinic visits and drug refills**	3–6 monthly clinic visits and drug refills.	Optimal frequency of clinical visits and refills for adults, children, and adolescents.
**Differentiated service delivery**	Reduced clinic visits for stable clients, package of care for advanced HIV disease.	Narrowing the choice of different models for stable clients (stable adults, children and adolescents, pregnant and breastfeeding women, key populations).
**Service integration**	HIV and TB treatment, ART provision where opioid substitution therapy is provided, integration of sexually transmitted infections and family planning services within HIV care settings, assessment and management of cardiovascular risk and depression.	HIV and noncommunicable diseases and sexual and reproductive health.
**Provider attitudes**	None.	Good practices to promote approachable and welcoming services.

ART, antiretroviral therapy; CBT, cognitive behavioral therapy; TB, tuberculosis.

Barriers to access to care, although critical, are beyond the scope of this review.

## Results

### Linkage from HIV testing to care

Globally, only 65% of HIV-positive individuals are receiving ART, and the proportion of HIV-negative individuals at high risk of HIV acquisition who have access to HIV prevention services is unknown [7]. Approximately a quarter of people living with HIV are unaware of their status; thus, a strategic mix of testing approaches is recommended, including facility-based and community-based testing strategies, self-testing, and assisted partner notification [8]. There is a renewed emphasis on ensuring that HIV testing is coupled with effective linkage to treatment and combination prevention services, supported by research and policies that promote starting treatment as soon as possible after an HIV-positive diagnosis is confirmed [9–11].

Updated WHO HIV testing guidelines, including recommendations on linkage to care, were released in December 2019. These guidelines emphasize that wherever HIV testing is provided, the person or organization doing the testing should be responsible for supporting clients and linking them to treatment and prevention services [12]. Effective linkage is particularly important when testing occurs outside of health facilities [13]. Current WHO guidelines emphasize several options for supporting timely linkage to care, including peer support and navigation, service integration (i.e., starting treatment across health services) and tracing of individuals who are not successfully linked [4]. Consideration of resources required for each approach could help prioritize future interventions.

Another potential strategy is to initiate ART in the community and then refer patients to the health facility for follow-up. A study from Lesotho found that people offered same-day ART start at home were more likely to link to care following an HIV-positive diagnosis than those referred to a health facility to initiate ART [14].

### Rapid initiation of ART

Research and program experience has shown that losses to care prior to starting ART can be reduced by reducing time to ART initiation [15,16]. WHO recommends rapid initiation of ART within 7 days following a confirmed HIV diagnosis and the offer of same-day ART initiation for those who are ready [17]. These recommendations have been adopted by most countries surveyed (S2 Text).

Rapid initiation of ART, including starting ART on the same day as HIV diagnosis, can lead to improved retention in care and increased viral suppression [18]. Concern regarding the possible risk of immune reconstitution inflammatory syndrome among people starting ART with advanced immunosuppression may lead to a delayed ART initiation. To minimize this risk, programs could improve baseline screening for advanced HIV disease, including tests to rapidly identify and treat, or exclude, active tuberculosis (TB) and cryptococcal meningitis without delaying ART initiation for most patients [19].

Some program reports suggest an increased risk of loss to care following ART initiation, highlighting the importance of focusing attention on adherence challenges during the initial period following ART initiation [16]. This is underscored by findings that good adherence in the initial months after starting ART initiation is predictive of long-term treatment success [20,21]. Ensuring that individuals receive adequate information to make an informed decision to start treatment is particularly important in the context of rapid ART initiation and same-day ART start.

Guidance is needed to identify the type, frequency, and duration of interventions that can improve treatment outcomes following rapid ART initiation, particularly for asymptomatic patients.

### Out-of-facility ART initiation

Community-based testing and rapid ART initiation have been implemented in several countries, but support is needed to ensure that ART is safely and successfully started outside of health facilities. A trial from Malawi found that more people started ART at home than those referred to a health facility [22]. Another trial, from Lesotho, found that more patients initiating same-day ART achieved viral suppression at 12 months (50%) than those referred to health centers (34%); acceptance of same-day ART on the same day was high, with 98% of patients indicating readiness [14]. A follow-up trial in Lesotho is underway to assess ART refills provided by village health workers [23], while another trial of community-based ART initiation is underway in South Africa and Uganda [24]. ART initiation outside of health facilities may also improve access to treatment for populations that may have difficulty accessing traditional health services: in Tanzania, community-based ART delivery for female sex workers improved ART initiation rates and reduced disengagement in care [25].

Out-of-facility ART initiation is another promising intervention for future WHO guidance. Benefits may vary according to context (e.g., HIV prevalence, urban versus rural setting, HIV testing strategy, and ART coverage). More research is needed to determine the optimal quantity of ART provided at initiation and the frequency and approach to ensure adherence counseling and appropriate clinical review, including laboratory testing and staffing needs. For patients with a CD4 cell count less than 200 cells/mm^3^, access to the WHO recommended package of care for advanced HIV needs to be improved, and consideration should be given to adapting the package to cover region-specific comorbidities [17].

### Task sharing and decentralization

Task sharing to support ART initiation by nonphysicians has been strongly recommended by WHO since 2013 and is supported by most national guidelines surveyed (S3 Text). However, this recommendation is generally limited to HIV testing and counseling and initiation of first-line ART for adults, with fewer guidelines recommending task sharing for ART initiation for children or initiation of second-line ART.

ART coverage remains low for children—52% as of the end of 2017 [26]. Task sharing could help ensure that lack of access to specialists does not hinder pediatric ART initiation unless clinically indicated. Similarly, up to 3 million people are estimated to need second-line ART in sub-Saharan Africa by 2020, but coverage is much lower [27]. Delaying or not switching regimens among people with confirmed virological failure has been partly attributed to lack of specialists required for starting second-line ART initiation [28]. Country initiatives to support task sharing for second-line ART initiation include a second-line prescriber exam for doctors, clinical officers, and nurses in Malawi and remote decision support from mentorship teams in Zimbabwe.

Task sharing also can support expanded access to key diagnostic interventions, including HIV testing and other simple diagnostic tests. WHO recommends HIV testing by lay providers [4], and task sharing of other diagnostic tests is broadly supported by a good practice statement in the WHO 2016 guidelines, which states that trained and supervised nonlaboratory staff, including lay people, can undertake finger-prick blood collection for testing [4]. Key essential diagnostic tests performed by lay providers could include point-of-care CD4 cell count, preparation of dried blood spot samples for early infant diagnosis and viral load (VL), point-of-care VL, cryptococcal antigen testing, and urine lateral flow lipoarabinomannan testing for TB at peripheral sites [17]. Implementing these recommendations can be supported by future guidelines that are more explicit about the range of interventions that can be undertaken by less-trained health workers.

A related recommendation is the decentralization of HIV treatment and care to peripheral health centers and community sites to increase the number of facilities providing ART, bring care closer to where people live, and decongest hospitals. Decentralization of ART to lower-level health centers has been widely adopted, but community ART provision remains limited (S4 Text).

### Antiretroviral delivery for stable clients

Since 2016, WHO has recommended reducing the frequency of clinic visits and ART refills for stable clients [4]. This approach to differentiated treatment delivery is supported by major funding agencies including The United States President’s Emergency Plan for AIDS Relief (PEPFAR) and the Global Fund and has been adopted by a number of national guidelines [29].

**Fig 1:**
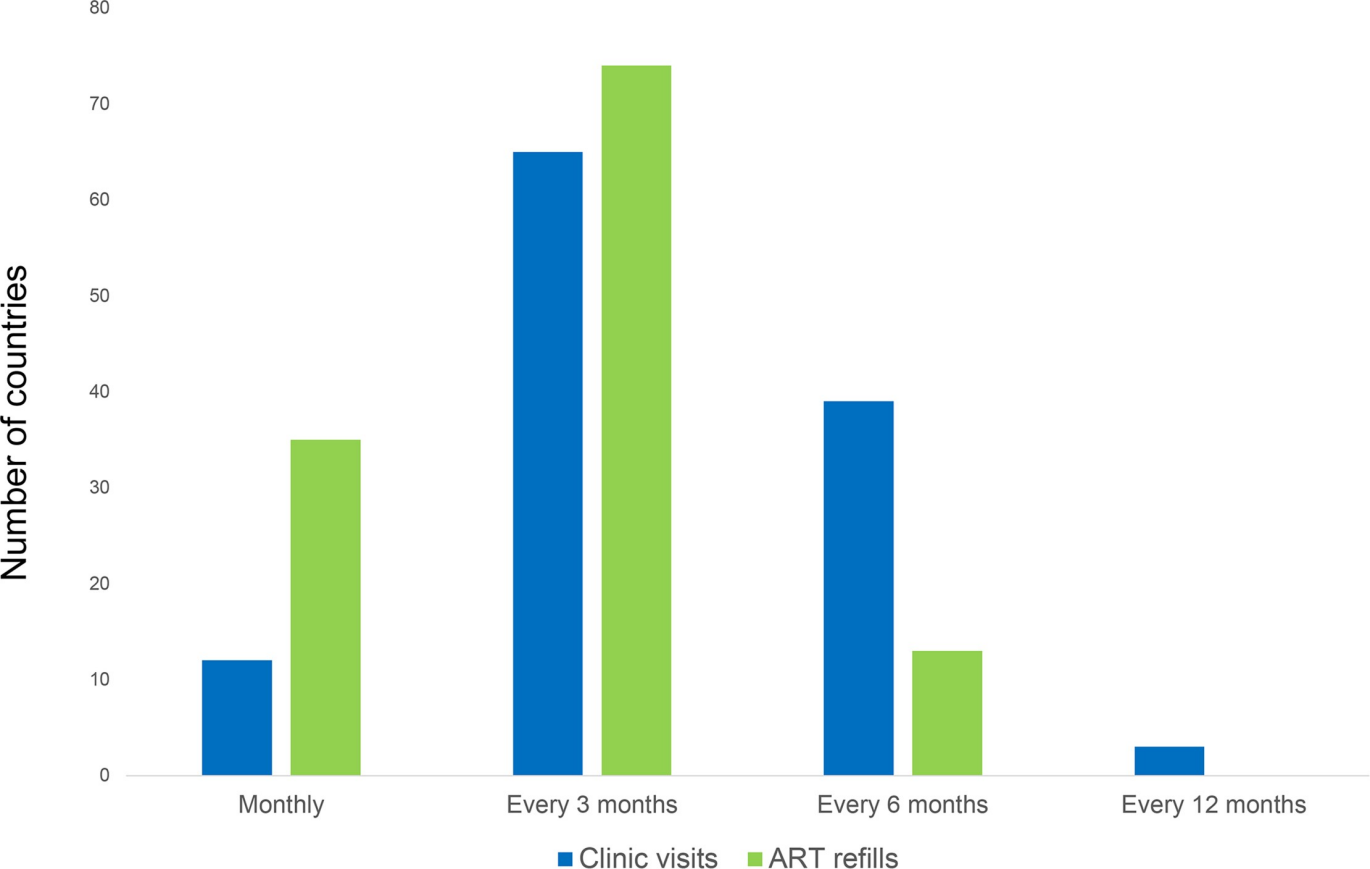
Country policies on the frequency of clinic visits and drug refills for people who are stable on ART. Data from UNAIDS, 2019: http://lawsandpolicies.unaids.org/ ART, antiretroviral therapy.

In 2016, WHO recommended reducing the frequency of clinic visits for stable ART clients to once every 3–6 months. Data from UNAIDS up to the end of 2019 show that of 120 countries surveyed, the majority (104) have adopted these recommendations (Fig 1).

A study from Zambia found that visits every 6 months were associated with decreased adverse outcomes (e.g., lateness or gaps in medication) and improved retention in care [30]. Some countries recommend annual clinical visits (e.g., South Africa and Zimbabwe); there are no data for the impact of annual visits on retention.

For children and adolescents, visits every 3 months are recommended more frequently in national guidelines than visits every 6 months, and several countries still require monthly visits for adolescents (S5 Text). Young children and adolescents are often evaluated monthly until they transition to adult doses, despite evidence and guidance that children aged ≥2 years may be seen every 3 months with drug dose adjustments managed appropriately [31,32].

The 2016 WHO guidelines also recommend ART refills once every 3–6 months for stable clients, and these recommendations have also been widely adopted by the majority of countries (Fig 1 and S5 Text). In Ethiopia, 6-month refills have been rolled out with initial positive findings [33]. A quality improvement intervention from Zambia increased the number of facilities offering 3-month refills in accordance with national policy, which resulted in 20 fewer patient visits per day in facilities receiving the intervention [34]. Since April 2019, following a Ministry of Health directive, Zambia also has scaled up 6-month refills for stable patients. Another study from Zambia found that patients preferred longer ART refill intervals—a 5-month interval was preferable to 3 months, which was preferable to 1 month [35,36].

Future guidance could help clarify the optimal frequency of clinic visits and ART refills. Policies could consider accommodating patient preferences and the need to simplify and align clinic visits and drug refills for patients with comorbidities.

### Integrating support for adherence and retention in care

Adherence to ART is critical to ensuring successful treatment outcomes and to reducing the risk of drug resistance. Although studies have suggested that 95% adherence is optimal, recent studies suggest that lower levels (80%–90%) may be sufficient with newer regimens [37].

VL is the preferred way to measure adherence, and viral suppression is high: above 80% among people retained in care [38]. Alternative approaches to assess adherence that are practical at scale in resource-limited settings include pharmacy refill records, self-reporting, and pill counts. Although there is currently no consensus on which approach provides the most reliable way to identify adherence challenges, pharmacy refill records are a feasible and reliable approach and are underutilized.

Peer counseling, adherence clubs, and mobile text messaging and reminder devices are recommended by current guidelines to support adherence and retention in care [4]. Reasons for suboptimal adherence are multiple and similar to reasons for disengagement from care, and some supportive interventions address both issues [39,40], suggesting that for future guidance interventions to support adherence, retention, and reengagement in care could be considered together as a single package.

### Peer support groups

Establishing peer support groups for stable ART patients both in health facilities and in the community fosters support and facilitates access to treatment [41]. Several models have been piloted and have improved outcomes for patients [42–46], including for children, adolescents, and breastfeeding women [47–49]. National HIV program managers prefer having a choice of models that can be applied in different contexts. Future guidance could propose limited options that would help national programs select the most appropriate model for differentiated service delivery given contextual factors, such as HIV prevalence, ART cohort size, health worker density, distance to health facilities, and comorbidities.

### Tracing and reengagement in care

Optimal approaches are needed to trace people who disengage from care and support return to care. Effective return to care includes rapid reinitiation of ART, clinical assessment, prophylaxis and management of opportunistic infections for patients with advanced HIV disease, and VL testing to assess need for regimen switch. Counseling also should be provided to understand reasons for disengagement and to offer any support required to facilitate return to care.

Tracing and reengagement programs can be effective [50,51]. A study from Uganda found that 70% of people traced after missing a scheduled clinic visit returned to care [51]. Another study from Kenya suggested that reengagement efforts are most effective when delivered in the initial period after disengagement [52]. Tracing also allows for clinics to track their outcomes more accurately and to use these outcomes for performance management.

Future guidance should recommend effective, feasible, and acceptable approaches to support tracing and reengagement with care and clarify who should perform the tracing and the type (e.g., by telephone or home visits) and frequency of interventions. Tracing should be prioritized for patients with high mortality risk, such as those who do not attend scheduled follow-up visits after hospital discharge [53].

A growing number of patients presenting with advanced HIV disease had previously started ART and then disengaged from care [54]. Guidance is needed to define what support should be provided after a period of prolonged disengagement from care, beyond the core package of care for patients with advanced HIV disease.

The use of national-level unique identifiers for patients would support effective tracing across the health system and improve return to HIV treatment as well as management for complex cases in decentralized clinical settings, via consultation or referral.

### Welcoming health services

A study from Zambia assessing patient preferences found that although longer ART refill intervals were preferred, patients were willing to accept shorter refills to access nice (as opposed to rude) providers [35]. This finding supports research from other studies that found that harsh and disrespectful treatment from health providers was a reason for disengagement for care [55–57].

Shame and fear of negative attitudes can lead people to be reluctant to return to care following disengagement [56]. In South Africa, clinics have piloted a successful “welcome back” counseling strategy that provides a nonjudgmental approach to reengagement in care [58]. Communicating acceptance also has encouraged reengagement in care in other settings [59]. Further research is needed to identify feasible, cost-effective approaches to improve health worker attitudes and patient experiences of care provision.

### Managing advanced HIV disease

WHO recommends a package of care for people presenting or re-presenting to care with advanced HIV disease [17]. Baseline CD4 cell counts should be obtained to identify patients with advanced HIV disease (defined as CD4 <200 cells/mm^3^). Patients with advanced HIV disease should be screened for TB and cryptococcus and provided with prophylaxis, given the high mortality rates associated with these coinfections. Future guidance should consider whether additional interventions should be included, especially to reduce the risk of mortality associated with severe bacterial infections [17,60] and to identify potentially serious coinfection at an earlier stage before symptoms develop [61].

### Service integration

Currently, WHO recommends service integration for common coinfections and comorbidities. These include providing both HIV and TB treatment, including TB preventive treatment in settings with a high burden of both diseases, providing ART in settings that offer opioid substitution therapy, integrating sexually transmitted infections (STIs) and family planning services within HIV care settings, and assessing and managing cardiovascular risk and depression [4]. Most countries surveyed offered integrated services for TB, maternal and child health, and STI services; integrating cancer screening and noncommunicable disease care was less common (S6 Text).

Several studies have assessed the impact of integrating care for HIV, hypertension, and diabetes [62], and integrating care for these conditions is recommended by some national guidelines. In Kenya, for example, blood pressure and glucose levels are monitored at baseline and follow-up, and clinicians providing ART are trained to manage diabetes and hypertension; basic screening for depression and alcohol and drug dependency is carried out routinely before ART initiation and yearly thereafter [63]. Malawi, South Africa, and eSwatini also have implemented policies for integrating HIV and noncommunicable disease care [63]. With the increasing scale-up of differentiated service delivery models for stable patients, care for other chronic diseases could be integrated with HIV services [64].

WHO recommends integrating family planning into ART services as a one-stop service, and future guidance could consider integrating family planning in differentiated service delivery models for stable clients. Many countries face persistent challenges, such as lack of supplies and trained health workers to providing long-acting, reversible contraception options such as implants and intrauterine devices. In Malawi, approximately 20% of women received family planning during ART consultations in 2018, and 69% of last pregnancies were unintended, which highlights the unmet need for family planning in women receiving ART. The high proportion of women who became pregnant while using contraception was ascribed to inconsistent condom usage or poor patient adherence to hormonal contraception [65]. In a partner-supported clinic in Malawi that integrated family planning into ART services, uptake of family planning increased from 28% to 62% [66].

Integration of HIV and noncommunicable diseases and sexual and reproductive health are key areas for future WHO guidance to support service integration. Service integration across a range of comorbidities should aim to align clinical follow-up and drug refill schedules as far as possible, adapted to the reduced frequency of contact with health services for the majority of clients who are stable on therapy.

## Discussion

Recommendations for HIV service delivery in WHO guidelines have been informed by evidence and experience of successful scale-up of ART across a range of settings and populations. Key areas requiring clarification within existing recommendations include task sharing and decentralization of services for pediatric populations and second-line ART prescription as well as prioritization of linkage to care interventions. New areas that require guidance include community-based ART initiation, patient tracing, and reengagement of people lost to follow-up.

HIV services could consider integrating the broad range of health needs of people living with HIV into models of care that may require less interaction with the health system while ensuring successful long-term outcomes of HIV treatment. Country experience of implementing differentiated service delivery models at scale has provided further evidence of the short-term impact of specific models for stable clients, those who may be at risk of advanced disease, and for specific populations. Ongoing evaluation would facilitate understanding of the net effects of introducing differentiated service delivery models on health service requirements and long-term outcomes for people living with HIV.

## Supporting information

S1 Text Country survey description. (DOCX)

S2 Text Rapid ART initiation and same-day start. ART, antiretroviral therapy.
(DOCX)

S3 Text Task sharing for ART initiation: Adults and adolescents. ART, antiretroviral therapy.
(DOCX)

S4 Text Decentralization of ART care: Adults and adolescents. ART, antiretroviral therapy.
(DOCX)

S5 Text Frequency of clinic visits and ART refills. ART, antiretroviral therapy.
(DOCX)

S6 Text Service integration. (DOCX)

## Abbreviations

ART: antiretroviral therapy
PEPFAR: President’s Emergency Plan for AIDS Relief
STI: sexually transmitted infection
TB: tuberculosis
UNAIDS: Joint United Nations Programme on HIV/AIDS
VL: viral load
WHO: World Health Organization
