# Supplementary material for: Emerging priorities for HIV service delivery
Source: PLoS Med. 2020 Feb 14;17(2):e1003028. doi: 10.1371/journal.pmed.1003028 (PMC7021280; doi:10.1371/journal.pmed.1003028)
Supplement: S1 Text — (DOCX) [file pmed.1003028.s001.docx]

**Supplementary File S1. Country Survey Description**

**Uptake of WHO recommendations for HIV Service Delivery: November 2018**

**Background**

In November 2018 WHO held an expert consultation on future priorities for HIV treatment service delivery. The consultation was attended by national HIV programme managers from 11 countries, implementation partners, representatives from civil society and academia.

To support this consultation, WHO conducted a survey to assess the implementation status of current guideline recommendations. The survey was sent by email to WHO country offices and directly to heads of national HIV programmes. Responses were received at the end of October 2018, from the following 17 countries: Botswana, Cameroon, Central African Republic, Cote d’Ivoire, eSwathini, Ethiopia, Ghana, Kenya, Lesotho, Mozambique, Myanmar, Pakistan, Rwanda, Sierra Leone, Vietnam, Zambia, and Zimbabwe.
