# Supplementary material for: Emerging priorities for HIV service delivery
Source: PLoS Med. 2020 Feb 14;17(2):e1003028. doi: 10.1371/journal.pmed.1003028 (PMC7021280; doi:10.1371/journal.pmed.1003028)
Supplement: S2 Text — ART, antiretroviral therapy. (DOCX) [file pmed.1003028.s002.docx]

**Supplementary File S2. Rapid ART initiation and same day start**
