# Supplementary material for: Emerging priorities for HIV service delivery
Source: PLoS Med. 2020 Feb 14;17(2):e1003028. doi: 10.1371/journal.pmed.1003028 (PMC7021280; doi:10.1371/journal.pmed.1003028)
Supplement: S3 Text — ART, antiretroviral therapy. (DOCX) [file pmed.1003028.s003.docx]

**Supplementary File S3. Task sharing for ART initiation: adults and adolescents**

**Task sharing for ART initiation: children**
