# Supplementary material for: Emerging priorities for HIV service delivery
Source: PLoS Med. 2020 Feb 14;17(2):e1003028. doi: 10.1371/journal.pmed.1003028 (PMC7021280; doi:10.1371/journal.pmed.1003028)
Supplement: S4 Text — ART, antiretroviral therapy. (DOCX) [file pmed.1003028.s004.docx]

**Supplementary File S4. Decentralization of ART care: adults and adolescents**

**Decentralization of ART care: children**
