# Supplementary material for: Emerging priorities for HIV service delivery
Source: PLoS Med. 2020 Feb 14;17(2):e1003028. doi: 10.1371/journal.pmed.1003028 (PMC7021280; doi:10.1371/journal.pmed.1003028)
Supplement: S6 Text — (DOCX) [file pmed.1003028.s006.docx]

**Supplementary File S6. Service integration**
